# Mediator Effect of Work Overload Between Fear of Future Violence at Work and Turnover Intention in Nurses

**DOI:** 10.1111/ijn.70145

**Published:** 2026-04-23

**Authors:** Ebru Dığrak, İrfan Akkoç, Yağmur Şenlier, Ezgi Başer

**Affiliations:** ^1^ Department of Nursing, Faculty of Health Sciences İzmir University of Economics Balçova İzmir Türkiye; ^2^ Rectorate İzmir Tınaztepe University İzmir Türkiye

**Keywords:** nurses, turnover intention, work overload, workplace sustainability, workplace violence

## Abstract

**Aim:**

Increasing rates of violence among healthcare workers contribute to increased fear of future violence, which consequently affects turnover intention. This study aimed to elucidate the mediating role of work overload on the relationship between fear of future violence at work and turnover intention in nurses.

**Methods:**

This explanatory cross‐sectional study was conducted with a sample of 124 nurses employed at a public hospital. Data were collected using a self‐administered survey that included a Personal Information Form, the Fear of Future Violent Events at Work Scale, the Work Overload Scale and the Turnover Intention Scale. Mediation analysis was performed using the PROCESS macro (Model 4) for SPSS with bootstrapping procedures to test indirect effects.

**Results:**

Results indicated a significant positive relationship between fear of future violence and work overload, as well as between work overload and turnover intention. While the total effect of fear of future violence on turnover intention was significant, the direct effect was not. Work overload significantly mediated the relationship between fear of future violence and turnover intention.

**Conclusion:**

The fear of future violence at work statistically significantly contributes to perceived increased workload, subsequently escalating the likelihood of turnover. These insights highlight the necessity for nursing policy interventions that aim to reduce work overload and address concerns related to future workplace violence. Creation of safer work environments may mitigate turnover intentions among nurses.

## Summary

What is already known about this topic?

- Workplace violence against nurses is prevalent globally and negatively affects both their psychological well‐being and job outcomes.
- Nurses experience fear of future violence even if they have not previously been subjected to violence.
- Work overload is an important predictor of nurses' turnover intention.

What this paper adds?

- Fear of future violence at work significantly increases nurses' perceived work overload.
- Work overload mediates the relationship between fear of future violence and turnover intention.
- Fear of future violence alone, even without direct exposure, can elevate nurses' turnover intention.

The implications of this paper:

- Healthcare institutions should implement policies to reduce fear of future violence and manage workload to retain nursing staff.
- Nursing management should address both actual and perceived threats to improve workforce stability and job satisfaction.
- Findings can guide interventions, policy‐making and future research aimed at preventing nurse turnover and mitigating workforce shortages.

## 1 Introduction

Nurses represent the largest professional group within the healthcare system and constitute a vital workforce that contributes to the improvement of health outcomes for individuals, families and communities through preventive and treatment interventions (Drennan and Ross 2019). Despite the nursing profession being recognized for its critical role in global health care, one of the most significant challenges faced today is the shortage of nurses (Tamata and Mohammadnezhad 2023). According to the World Health Organization (WHO) (2020), there are approximately 27.9 million nurses globally, along with a projected shortage of 5.9 million nurses, which is expected to reach 10.6 million by 2030. Furthermore, between 4% and 54% of nurses worldwide are considering leaving the profession (WHO 2016), while in Türkiye, this figure ranges from 35.3% to 50.6% (Sabanciogullari and Dogan 2015; Aydoğmuş and Özlük 2022). Nurses' turnover not only leads to disruptions in healthcare services but also results in the loss of valuable skills and expertise within the nursing field. This situation jeopardizes the continuity and quality of patient care, creating negative impacts on patient outcomes and the overall delivery of healthcare services (Lu et al. 2019).

The increasing turnover intention among nurses worldwide is based on numerous complex reasons. Research indicates that nurses consider leaving their jobs due to preventable factors such as job satisfaction, work environment, burnout, salary, career satisfaction, workload and violence in healthcare settings (Seki and Özlük 2024; Turunç et al. 2024; Alkan et al. 2024; Shin et al. 2018; Phillips 2020; Kang et al. 2020). While these issues are widespread globally, they vary from country to country due to differences in healthcare systems, socioeconomic structures and cultural characteristics. Although there are studies that aim to understand the variables influencing nurses' decisions to leave their jobs, gaps still exist in the literature regarding some of these variables. This study will focus on the variables of workload and the fear of future violence at work.

**FIGURE 1:**
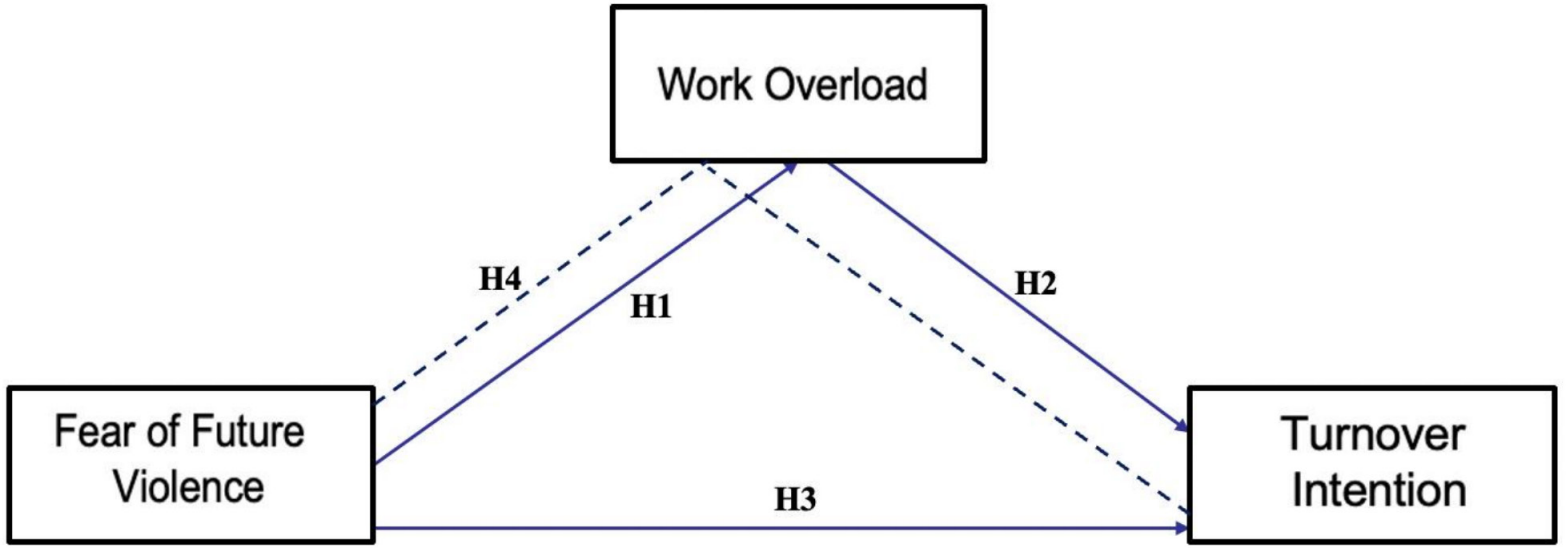
Structural equation of model.

As far as we know, this study is the first to examine these three variables concurrently among nurses. Additionally, it is the first research to address the mediating role of work overload in the impact of fear of future violence at work on turnover intention. In this context, the aim of the study is to investigate the mediating effect of work overload on the relationship between fear of future violence at work and turnover intention among nurses. This model is expected to provide new insights into the factors leading to nurses' turnover and to be effective in improving the functioning of healthcare institutions. In this regard, the model of the study is presented in Figure 1.

The hypotheses of the study have been established as follows to evaluate the relationships between these concepts.

### 1.1 Fear of Future Violence at Work and Work Overload

Workplace violence refers to situations that involve harassment, threats or assault at work (Sheikhbardsiri et al. 2022). Violence directed at the healthcare sector has reached statistically significant levels globally and has become a critical public health issue that threatens the resilience of health system (Kuhlmann et al. 2023). The World Health Organization (2022) reported that healthcare workers are increasingly facing threats or verbal assaults, with between 8% and 38% experiencing physical violence at some point in their careers. Among healthcare professionals, nurses are particularly at risk due to their proximity to patients and visitors (Zhang et al. 2023; Li et al. 2020; WHO 2022). Recent systematic reviews and meta‐analyses have shown that the prevalence of violence against nurses by patients and visitors is alarmingly high (Sahebi et al. 2022; Zhang et al. 2023). One report indicates that one in four nurses has reported being assaulted at some point in their careers, while one in two nurses states that they experience an assault every hour (American Nurses Association 2021). Furthermore, it is believed that the existing statistics do not accurately reflect the true extent of the problem due to underreporting among nurses (Spencer et al. 2023). Additionally, nurses fear being subjected to violence in the future due to the prevalence of workplace violence and the unsafe working conditions (Shahzad and Malik 2014). This has resulted in the recognition of the fear of future violence at work as a significant issue.

The fear of future violence at work encompasses individuals' fears about experiencing both physical (e.g., hitting, kicking, holding, pushing and biting) and nonphysical forms of violence (e.g., threats involving weapons and verbal harassment) that they may encounter in the workplace (Güneş et al. 2024). This fear can be characterized as an emotional response to the perceived risk of future violence (Portoghese et al. 2017). Some studies indicate that nurses experience this fear, even if they have not been subjected to violence throughout their careers (Fu, Wang, et al. 2021; Fu et al. 2023; Akbolat et al. 2021; Pacheco et al. 2022; Güneş et al. 2024). The fear experienced by nurses negatively impacts their work performance and contributes to work overloads (Pacheco et al. 2022).

In nursing, workload refers to the amount of care a nurse is able to provide to patients. It encompasses various activities such as administrative tasks, training nursing students, attending meetings and engaging in professional development (Alghamdi 2016). Work overload implies that nurses must work faster and under time pressure due to an overwhelming amount of work assigned to each individual (Ilies et al. 2015). The fear of future violence at work impacts nurses' performance and increases the likelihood of experiencing difficulties in managing their workload (Fu, Wang, et al. 2021; Barbe et al. 2018). In this context, stress and the fear of future violence at work may contribute to an increase in work overload among nurses. Currently, there is no research in the literature that examines the direct relationship between work overload and fear of future violence at work. Based on the previously mentioned literature, Hypothesis 1 has been formulated on the premise that work overload may positively and significantly influence the fear of future violence at work.

Hypothesis 1 *Fear of future violence at work affects work overload*.

### 1.2 Work Overload and Turnover Intention

The increasing workload for nurses may necessitate working longer hours than their regular shifts, leading to both physical and mental fatigue (Arifiani et al. 2019). An increase in perceived workload can negatively affect nurses' professional and personal lives, potentially resulting in a greater intention to leave their jobs. Turnover intention refers to the process of contemplating, planning and deciding to quit a job or profession. Although turnover intention does not always lead to actual turnover, it represents a significant step in that direction (Chao et al. 2015). A study conducted by Said and El‐Shafei (2021) reported that 98.6% of nurses identified workload as one of the most significant factors influencing their intention to leave. Moreover, research conducted in various countries has demonstrated that work overload increases nurses' turnover intentions (Phillips 2020; Halter et al. 2017). Consequently, Hypothesis 2 has been formulated.

Hypothesis 2 *Work overload affects turnover intention*.

### 1.3 Fear of Future Violence at Work and Turnover Intention

Recent studies indicate that the prevalence of workplace violence experienced by nurses is significantly high in many countries, including Turkey (Pariona‐Cabrera et al. 2020; Demirci and Uğurluoğlu 2020). This violence affects their safety, health and overall well‐being, both directly and indirectly (Kumari et al. 2022). Furthermore, nurses are negatively impacted not only by the actual violence they experience but also by the fear of future violence at work (Akbolat et al. 2021). For instance, the fear of future violence at work can lead to depressive symptoms among nurses (Fu et al. 2023), emotional exhaustion and cynicism (Portoghese et al. 2017), as well as burnout (Fu, Ren, et al. 2021). It has been found that the negative emotions arising from this fear, specifically the fear of future violence, increase the intention to leave their jobs (Akbolat et al. 2021). Consequently, it is hypothesized that the fear of future violence at work may influence turnover intention, leading to the formulation of Hypothesis 3.

Hypothesis 3 *Fear of future violence at work affects turnover intention*.

### 1.4 Work Overload, Fear of Future Violence at Work and Turnover Intention

The previously mentioned literature provides evidence that work overload may act as a mediating factor in the relationship between turnover intention and the fear of future violence at work. Various studies have demonstrated that work overload has a mediating effect on the relationships among different variables (Jiandong et al. 2022; Wang 2022). Additionally, it has been found that work overload mediates the influence of various factors on turnover intention (Çelik and Çıra 2013). However, no current research examines the mediating role of work overload in the correlation between fear of future violence at work and turnover intention. Therefore, based on the existing literature, Hypothesis 4 has been formulated under the assumption that work overload will act as a mediating factor.

Hypothesis 4 *Work overload is a mediator between fear of future violence at work and turnover intention*.

## 2 Methods

### 2.1 Study Design

This study was designed as an explanatory, analytical cross‐sectional study using mediation analysis to investigate the relationships among fear of future violence at work, work overload and turnover intention. Using survey design, this evaluation began by assessing the direct impact of the independent variables on the mediator variable, followed by an analysis of the direct influence of the independent variable on the dependent variable. Lastly, the effect of the mediator variable on the dependent variable was scrutinized while controlling for the independent variable.

### 2.2 Sampling and Participants

The study population consisted of 180 nurses working at a public hospital between February and June 2023. Based on a 95% confidence level and a 5% margin of error, the required sample size was calculated as 123 (Israel 2013; Sekaran and Bougie 2016). Participants were selected using a convenience sampling method. Inclusion criteria were being actively employed as a nurse, having at least 1 year of professional experience, and providing voluntary consent to participate in the study. A total of 124 nurses who met the inclusion criteria and agreed to participate completed the study.

### 2.3 Instruments

To collect the research data, the following four scales were used: Personal Information Form, Fear of Future Violent Events at Work Scale, Work Overload Scale and Turnover Intention Scale.

Personal Information Form, created by the researchers, consists of seven sociodemographic questions concerning age, gender, marital status, educational status, years of work experience, department and shift characteristics. Participants were asked to report the total number of shifts they worked per month. In the institution, shifts are 16 h in duration, and evening–night shifts cover the period from 16:00 to 08:00.

#### 2.3.1 The Fear of Future Violent Events at Work Scale

This scale was developed by Rogers in 1994 to measure future fear of violence in the workplace, with an original Cronbach's alpha value of 0.94 (Rogers 1994). The scale consists of a single dimension and employs a 5‐point Likert scale (1 = *Strongly Disagree*, 5 = *Strongly Agree*). It includes 10 statements related to physical (e.g., shoving, hitting, biting and kicking) or nonphysical (e.g., verbal abuse and threats) violence that participants may be exposed to or fear experiencing in the upcoming year at work (e.g., ‘I am afraid of becoming a victim of workplace violence’). A high average indicates that participants perceive a greater fear of being subjected to violence from patients and their relatives within a short time frame (1 year). The Turkish validity and reliability study of the scale was conducted among healthcare workers by Akbolat et al. (2021), which found a Cronbach's alpha value of 0.94. Similarly, in the country where this study was conducted, the Cronbach's alpha value for nurses reported by Güneş et al. (2024) was 0.94, whereas this study calculated it to be 0.95.

#### 2.3.2 Work Overload Scale

This scale was developed by Duxbury and Higgins (1991) to assess the work overload experienced by employees due to their work environment. The scale consists of 11 items and is unidimensional (e.g., ‘My work requires me to work long hours’). It employs a 5‐point Likert scale, allowing respondents to score their answers on a scale of 1 to 5 (1 = *Strongly Disagree*, 5 = *Strongly Agree*). As the score from the scale increases, it indicates a higher level of workload resulting from the work environment. A Turkish validity and reliability study was conducted by Aycan and Eskin (2005), which found a Cronbach's alpha reliability coefficient of 0.84. Additionally, a study conducted by Seki and Özlük (2024) among nurses in the same country found a reliability coefficient of 0.87, whereas this study calculated the reliability coefficient to be 0.92.

#### 2.3.3 Turnover Intention Scale

This scale was developed by Wayne et al. (1997) to determine individuals' intentions to leave their jobs, and it has a reliability coefficient of 0.89 reported in their study. This scale is a unidimensional, five‐item measure using a 5‐point Likert scale (1 = *Strongly Disagree*, 5 = *Strongly Agree*). High scores on the scale indicate a strong intention to leave among individuals. A Turkish validity and reliability study conducted by Avcı and Küçükusta (2009) found that the reliability coefficient of the translated scale was 0.72. Additionally, a study conducted by Turunç et al. (2024) among nurses in the same country found a reliability coefficient of 0.81, while analysis in this study calculated it to be 0.95.

### 2.4 Data Collection

Data were collected between February and June 2023 at the hospital using a self‐administered questionnaire. Prior to data collection, nurses were informed about the purpose of the study, and both written and verbal consent were obtained. Nurses who met the inclusion criteria were invited to participate. Data were collected through face‐to‐face contact during nurses' self‐identified break periods by the researchers. Completion of the questionnaire took approximately 10–15 min.

### 2.5 Statistical Analysis

The data were processed using the Statistical Package for the Social Sciences (Version 22), with the statistical significance threshold set at a 95% confidence interval. In the preliminary stage, we determined the congruity of the measurement model with the data. A confirmatory factor analysis (CFA) was conducted to assess the structural validity of the research model, utilizing the maximum likelihood estimation method. To ensure the robustness of our model, we meticulously evaluated the fit of the hypothesized three‐factor framework, which included fear of future violence at work, turnover intention, and work overload. This evaluation involved various fit indices, including *χ* ^2^/df (degree of freedom), root mean square error of approximation (RMSEA), comparative fit index (CFI), and standardized root mean squared residual (SRMR). Additionally, *χ* ^2^ difference tests provided further insights (Kline 2016). As a benchmark, we considered fit values of *χ* ^2^/df < 5, RMSEA < 0.08, CFI > 0.90, and SRMR < 0.08 to indicate acceptable model conformity (Hu and Bentler 1999). The reliability analysis of the scales was facilitated by Cronbach's alpha coefficient.

Descriptive statistics were calculated to delineate the sociodemographic characteristics of the participants. Pearson correlation analysis was used to examine correlations among the study variables, specifically fear of future violence at work, turnover intention and work overload.

Following these preliminary analyses, the mediation model was examined using AMOS and the PROCESS macro for SPSS. Hayes' (2022) bootstrapping method (Model 4) was employed to test the mediation relationships. Total, direct, and indirect effects were estimated using 5000 bootstrap resamples with both percentile and bias‐corrected 95% confidence intervals. The primary aim was to determine the indirect effect of work overload as the mediator. According to Hayes (2022), mediation is considered statistically significant when the 95% bootstrap confidence interval for the indirect effect (BootLLCI and BootULCI) does not include zero.

### 2.6 Ethical Considerations

This study was approved by İzmir University of Economics Health Sciences Research Ethics Committee (05‐20‐195, 19/12/2022). In addition, permission was obtained from the institution where the study was conducted. Prior to participation, nurses were informed about the study aims and procedures both face to face and through a written information sheet, and written informed consent was obtained from all participants. The survey text indicated that participants were not obliged to participate in or submit the survey. If a participant did not complete the consent form, they were excluded from the study.

## 3 Results

### 3.1 Test for Common Method Bias and Measurement Validation

Given the susceptibility of cross‐sectional datasets to common method bias, we initially employed Harman's one‐factor test to assess its presence. In the exploratory factor analysis, all the items measuring the construct were constrained to load onto a single factor. The analysis revealed that one component explained 38.23% of the variance, which was below the 50% threshold (Podsakoff et al. 2012), indicating that common method bias was not a significant concern.

We conducted a confirmatory factor analysis (CFA) using maximum likelihood estimation within the AMOS 21 software to assess the extended psychometric properties of the instruments used. The results of the CFA indicated an acceptable fit for the measurement model, as demonstrated by the following fit metrics: *χ* ^2^(269, *N* = 364) = 772.43; *p* < 0.001; *χ* ^2^/df = 2.87; RMSEA = 0.07; CFI = 0.92; SRMR = 0.05. All standardized loading estimates were statistically significant, expert for two estimates related to the ‘turnover intention’ construct, which were 0.5. One item from this construct was removed due to a loading estimate below 0.40, which negatively affected the construct's average variance extracted (AVE) by maintaining it below the 0.50 criterion. Following this omission, the CFA results indicated that the AVE for all constructs exceeded the recommended threshold of 0.50, providing evidence of convergent validity (Hair et al. 2013). As shown in Table 2, the composite reliability for each construct exceeded 0.70, further supporting the claim of convergent validity (Fornell and Larker 1981). To evaluate the discriminant validity of the constructs, the authors utilized the average variance extracted (AVE) methodology. Table 2 shows that for each pair of constructs, the AVE exceeds the respective squared correlation, supporting the case for discriminant validity in accordance with Fornell and Larker's (1981) guidelines.

### 3.2 Participant Characteristics and Correlational Findings

The demographic profile of the study participants indicated that 96% of the nurses were female, 71.8% were married, and 69.4% held undergraduate qualifications. The average age of the nurses was 39.33 ± 8.37 years, with ages ranging from 22 to 62 years. The average years of experience among nurses was 17.81 ± 9.27 years, with 41.1% working in medical wards and 28.3% in outpatient clinics (Table 1).

**TABLE 1:** Demographic and work‐related characteristics of participants (*N* = 124).

Variables	Category	*N*	%
Age		M = 39.33	SD = 8.37
Gender	Female Male	119 5	96.0 4.0
Marital status	Married Single	89 35	71.8 28.2
Educational status	Below undergraduate Undergraduate Master or above	16 86 22	12.9 69.4 17.7
Working years		M = 17.81	9.27
Department	Medical wards Outpatient clinics Emergency Intensive care unit	51 35 19 19	41.1 28.3 15.3 15.3
Shift work	Yes No	88 36	71.0 29.0
Number of shifts (per month, 16‐h shifts)	1–3 4–6 7–9 10–12	17 36 20 15	19.3 41.0 22.7 17.0

Abbreviations: % = percentage; M = mean; *N* = number; SD = standard deviations.

Fear of future violence at work (*r* = 0.38, *p* < 0.01), and work overload (*r* = 0.30, *p* < 0.01) were positively correlated with turnover intention, indicating that increasing fear of future violence and work overload in hospitals is associated with heightened turnover intention among nurses. In the demographic data, the work overload score showed a negative correlation with average age (*r* = −0.19, *p* < 0.05), suggesting that as the age of nurses increased, their workload tended to decrease. These findings provide initial support for the hypotheses outlined in this study (Table 2).

**TABLE 2:** Reliability and Pearson's correlations among study variables (*N* = 124).

Variables	AVE	CR	*α*	M	SD	1	2	3	4	5	6
1. Fear of future violence	0.66	0.95	0.95	3.42	1.12						
2. Work overload	0.60	0.92	0.92	4.19	0.76	0.38**					
3. Turnover intention	0.82	0.95	0.95	2.83	1.31.	0.30**	0.37**				
4. Age				39.33	8.37	0.01	−0.19*	−04			
5.Gender (1 = female, 2 = male)						0.13	−0.01	0.08	−31**		

Abbreviations: AVE = average variance extracted; CR = composite reliability; M = mean; SD = standard deviation; *α* = Cronbach's alpha.

* *p* < 0.05.

** *p* < 0.01(two‐tailed).

### 3.3 Assessing Direct and Indirect Effects in the Proposed Mediation Framework

**FIGURE 2:**
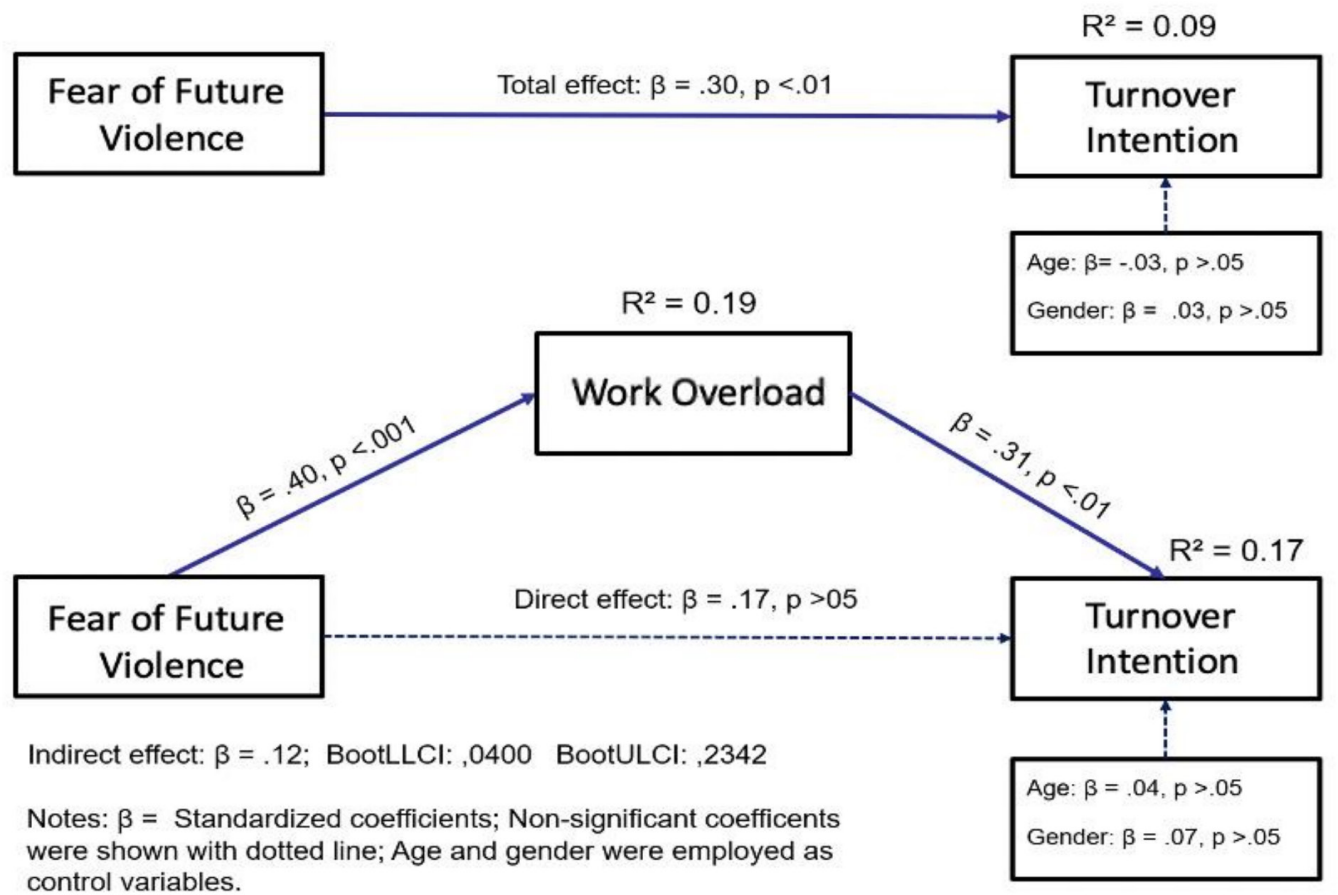
Summary of bootstrapping results.

To evaluate the hypothesized relationships, the proposed mediation framework was examined through an analysis of both direct and indirect effects. Table 3 and Figure 2 present the path analysis results, which showed that fear of future violence at work had a significant and positive effect on work overload (*β* = 0.40, *p* < 0.01), supporting Hypothesis 1. Overall, the model explained 19.33% of the variance in work overload (*R* ^2^ = 0.19, *F* = 9.58, *p* < 0.001).

**TABLE 3:** Path analysis results and total, direct and indirect effects in model (*N* = 124).

Outcome variable: Work overload						
	*β*	se	*t*	*p*	LLCI	ULCI
Fear of future violence	0.3955	0.0563	4.7770	0.0000	0.1574	0.3802
Age	−0.2308	0.0078	−2.6747	0.0085	−0.0365	−0.0054
Gender	−0.1330	0.3350	−1.5277	0.1292	−1.1749	0.1515
*R* = 0.4396; *R* ^2^ = 0.1933; *F* = 9.5827***						
Outcome variable: Turnover intention						
	*β*	se	*t*	*p*	LLCI	ULCI
Fear future of violence	0.1737	0.1075	1.8896	0.0612	−0.0097	0.4159
Work overload	0.3117	0.1599	3.3556	0.0011	0.2199	0.8530
Age	0.0393	0.0141	0.4347	0.6645	−0.0218	0.0341
Gender	0.0743	0.5923	0.8311	0.4076	−0.6805	1.6650
*R* = 0.4144; *R* ^2^ = 0.1717; *F* = 6.1683***						
Fear of future violence → turnover intention						
	*β*	se	*t*	*p*	LLCI	ULCI
Total effect	0.2970	0.1026	3.3831	0.0010	0.1440	0.5505
Age	−0.0051	0.0143	−0.3569	0.7218	−0.0334	0.0232
Gender	0.2177	0.6111	0.3563	0.7223	−0.9923	1.4278
*R* = 0.3055; *R* ^2^ = 0.0934; *F* = 4.1188**						
Fear of future violence → turnover intention						
	*β*	se	*t*	*p*	LLCI	ULCI
Direct effect	0.1737	0.1075	1.8896	0.0612	−0.0097	0.4159
Fear of future violence → work overload → turnover intention						
	*β*	BootSE	BootLLCI	BootULCI		
Indirect effect	0.1233	0.0498	0.0400	0.2342		

*Note:* standardized coefficients are reported; age and gender were controlled; bootstrap sample size = 5000.

Abbreviations: *β* = regression coefficient; LLCI = 95% lower limit of confidence; se = standard error; ULCI = 95% upper.

** *p* < 0.01.

*** *p* < 0.001 (two‐tailed).

When turnover intention was examined as the outcome, work overload showed a significant and positive effect (*β* = 0.31, *p* < 0.01), supporting Hypothesis 2. In contrast, the direct effect of fear of future violence on turnover intention was not statistically significant (*β* = 0.17, *p* = 0.06). The model accounted for 17.17% of the variance in turnover intention (*R* ^2^ = 0.17, *F* = 6.17, *p* < 0.001).

In the total‐effect model, where the mediator was omitted, fear of future violence demonstrated a significant effect on turnover intention (*β* = 0.30, p < 0.01), supporting Hypothesis 3. This model explained 9.34% of the variance in turnover intention (*R* ^2^ = 0.09, *F* = 4.12, *p* < 0.01).

Finally, the mediation analysis indicated that work overload played a significant mediating role in the relationship between fear of future violence and turnover intention. The 95% bootstrap confidence interval for the indirect effect did not include zero (*β* = 0.12, BootSE = 0.05, BootLLCI = 0.0400, BootULCI = 0.2342), thereby supporting Hypothesis 4.

## 4 Discussion

This study aimed to examine the effect of fear of future violence on turnover intention among nurses working in a state hospital, as well as how work overload mediates this effect. The research findings revealed complex relationships between fear of future violence at work, turnover intention and work overload, providing a deeper understanding of the factors affecting nurses' retention within the healthcare system and offering new contributions to the literature.

First, fear of future violence at work has a positive effect on nurses' work overloads (Hypothesis 1). The existing literature reports that as the incidence of violence experienced by nurses increases, so do their levels of workload (Huang et al. 2020; Havaei and MacPhee 2020). Another study has found that the fear of future violence at work has a negative impact on healthcare workers' performance and skills (Ugan and Akbolat 2023). This situation can be linked to the increasing workload faced by nurses. However, to our knowledge, the relationship between the fear of future violence and work overload has not been investigated, further emphasizing the significance of this study.

Second, our findings indicate that work overload positively influences the turnover intention among nurses (Hypothesis 2). This result is consistent with existing literature that underscores the relationship between work overload and turnover intention (Boamah et al. 2017; Lee et al. 2020; Phillips 2020; Halter et al. 2017). The turnover intention is recognized as a significant predictor of actual turnover among nursing staff. When nurses leave their positions or the profession entirely, it often results in increased workloads for the remaining staff, which can have detrimental effects on the overall healthcare system. Therefore, these findings further highlight the necessity of effectively managing work overloads to ensure sustainability within the nursing profession and to enhance the quality of healthcare services.

Third, this study demonstrates a positive relationship between fear of future violence at work and the turnover intention the nursing profession (Hypothesis 3). Research and meta‐analyses focusing on nurses indicate that workplace violence significantly affects their turnover intention and has a direct impact on turnover rates (Pang et al. 2023; Gedik et al. 2023; Jang et al. 2022; Stafford et al. 2022). Additionally, various meta‐analyses have reported that lateral violence among nurses, as well as ridicule, criticism and verbal harassment from doctors and patients, are primary reasons for nurses' turnover (Zhang et al. 2022; Lee and Kang 2018). While it is well established in the literature that nurses who have experienced violence tend to have turnover intentions their jobs, this study makes a novel contribution by demonstrating that the fear of future violence itself can also trigger a turnover intention among nurses. Although no previous studies have specifically examined the relationship between fear of future violence at work and turnover intention among nurses, a similar study conducted among healthcare workers has shown a positive effect of fear of future violence on the turnover intention (Akbolat et al. 2021). This finding supports our research. In conclusion, regardless of whether nurses have experienced workplace violence, it is evident that fear of future violence has detrimental effects and increases their turnover intention. This situation underscores the critical importance of addressing fear of future violence as a significant issue within the healthcare sector and highlights the need for effective solutions.

Lastly, the findings indicate that work overload acts as a mediating variable in the relationship between fear of future violence at work and the turnover intention in the profession (Hypothesis 4). Previous research conducted across various occupational groups has examined work overload as a mediating factor in the relationships among burnout, organizational citizenship behaviour, chronic anxiety and turnover intention (Akçakanat and Uzunbacak 2019; Rotenstein et al. 2023; Çelik and Çıra 2013). Specifically, among nurses, work overload has been explored as a mediating variable in the context of various factors, including role conflict, role ambiguity and workplace harassment (Akbolat et al. 2022). However, to our knowledge, this study provides the first scientific evidence that fear of future violence at work influences nurses' turnover intention, with work overload serving as a mediating variable. The practical implications of this study emphasize the critical roles that fear of future violence and work overload play in turnover intention, highlighting the necessity of including these three variables in interventions aimed at reducing nurses' turnover intention in the profession.

### 4.1 Limitations

The research findings contribute to the existing literature, but it is important to acknowledge several limitations. First, the use of a cross‐sectional model complicates the examination of correlations between variables and makes it difficult to establish causality. Additionally, our study was conducted in Türkiye, which may limit the generalizability of the findings. Future researchers may find it beneficial to investigate the fear of future violence in healthcare systems across diverse regions, where prevalence rates may vary. Moreover, our study focused specifically on nurses' turnover intentions rather than their actual turnover behaviours. Future research could explore the decision‐making processes of nurses who have turnover intention but ultimately choose to stay. A comprehensive investigation into this topic could yield deeper insights into the factors influencing nurses' turnover decisions. The data for this study were collected from nurses working in a public hospital located in an urban centre; therefore, the findings may not represent perspectives from private or rural healthcare settings. Furthermore, while it was assumed that nurses provided sincere and accurate responses to the measurement instruments, this may not always have been the case. To enhance our understanding of the relationships between fear of future violence at work, work overload and turnover intentions, it is recommended that future studies address these limitations and explore these dynamics in greater depth.

## 5 Conclusions

This study provides new evidence regarding the impact of fear of future violence on nurses' turnover intention in the profession, highlighting the mediating role of work overload in this relationship. Fear of future violence has led to an increase in workload, which negatively influences the turnover intention. Additionally, the findings demonstrate that nurses experience significant fear of future violence at work. This underscores the importance of identifying not only those who have been subjected to violence but also those who live in fear, necessitating the implementation of appropriate measures. Fear of future violence is a critical factor influencing nurses' decisions to leave the profession, which poses a concern on a global scale. To address the fear of future violence among nurses, it is essential to establish policies that include the enforcement of strict penalties for acts of violence, an increase in the number of security personnel, ongoing monitoring of nurses' psychological health and the implementation of preventive measures. These strategies are anticipated to effectively reduce turnover intentions in the profession. Moreover, alleviating fear of future violence is likely to enhance job satisfaction and potentially reduce turnover rates, thereby improving the overall effectiveness and efficiency of the nursing profession. Furthermore, this study will serve as a guide in identifying factors that contribute to nurse shortages. In conclusion, this research emphasizes the necessity for nursing management to proactively address the impacts of workplace violence, fear of future violence and the resultant work overload.

Finally, the lack of published research in this area highlights the importance of future studies. It is recommended that subsequent research focus on the individual and institutional factors influencing nurses' fear of future violence, as this will provide a deeper understanding of this critical issue.

This study is closely aligned with the United Nations Sustainable Development Goals, particularly SDG 3: Good Health and Well‐Being and SDG 8: Decent Work and Economic Growth. Fear of future workplace violence, excessive workload and turnover intention among nurses is a critical issue that directly affects the safety, well‐being and sustainability of the healthcare workforce. In addition, given that the nursing profession is predominantly female, the study also indirectly contributes to SDG 5: Gender Equality by addressing the safety and well‐being of women in the workplace (United Nations 2015).

## Author Contributions

**Ebru Dığrak:** conceptualization (equal), methodology (supporting), formal analysis (supporting), software (lead), data curation, investigation, writing – original draft preparation (lead), writing – review and editing (lead). **İrfan Akkoç:** conceptualization (lead), methodology (lead), formal analysis, software, data curation, writing – review and editing (supporting). **Yağmur Şenlier:** conceptualization (supporting), writing – review and editing (supporting). **Ezgi Başer:** conceptualization (supporting), writing – review and editing (supporting).

## Conflicts of Interest

The authors declare no conflicts of interest.

## Funding

The authors have nothing to report.

## Data Availability

The data that support the findings of this study are available from the corresponding author upon reasonable request.
